# Unraveling the Dynamics of SARS-CoV-2 Mutations: Insights from Surface Plasmon Resonance Biosensor Kinetics

**DOI:** 10.3390/bios14020099

**Published:** 2024-02-13

**Authors:** Devi Taufiq Nurrohman, Nan-Fu Chiu

**Affiliations:** 1 Laboratory of Nano-Photonics and Biosensors, Institute of Electro-Optical Engineering, National Taiwan Normal University, Taipei 11677, Taiwan; 81077004h@ntnu.edu.tw; 2 Department of Life Science, National Taiwan Normal University, Taipei 11677, Taiwan

**Keywords:** COVID-19, surface plasmon resonance, biosensors, kinetics

## Abstract

Surface Plasmon Resonance (SPR) technology is known to be a powerful tool for studying biomolecular interactions because it offers real-time and label-free multiparameter analysis with high sensitivity. This article summarizes the results that have been obtained from the use of SPR technology in studying the dynamics of severe acute respiratory syndrome coronavirus 2 (SARS-CoV-2) mutations. This paper will begin by introducing the working principle of SPR and the kinetic parameters of the sensorgram, which include the association rate constant (k_a_), dissociation rate constant (k_d_), equilibrium association constant (K_A_), and equilibrium dissociation constant (K_D_). At the end of the paper, we will summarize the kinetic data on the interaction between angiotensin-converting enzyme 2 (ACE2) and SARS-CoV-2 obtained from the results of SPR signal analysis. ACE2 is a material that mediates virus entry. Therefore, understanding the kinetic changes between ACE2 and SARS-CoV-2 caused by the mutation will provide beneficial information for drug discovery, vaccine development, and other therapeutic purposes.

## 1. Introduction

The global coronavirus disease 2019 (COVID-19) pandemic, caused by the SARS-CoV-2 virus, has resulted in many deaths and severe economic problems in all countries around the world [1,2,3]. To date, SARS-CoV-2 has mutated thousands of times, and these mutations occur spontaneously during replication [4]. These mutations will have direct implications with the increasing number of new variants of the virus. The bad news is that new variants of SARS-CoV-2 can affect the transmission rate of the virus, the severity of the disease, and the efficacy of the vaccine [5]. The development of sensitive and accurate analytical technology is significant for the purpose of screening and handling viruses that have a high level of spread and transmission that is difficult to avoid. The standard methods have been used to detect COVID-19, including RNA detection by reverse transcription polymerase chain reaction (RT-PCR) from respiratory samples [6], antibody detection by the enzyme-linked immunosorbent assay (ELISA) [7], chest X-rays [8], and computed tomography [9].

Of the several methods mentioned, RT-PCR is still the gold-standard clinical diagnostic method for detecting COVID-19 [10]. Unfortunately, this method requires special equipment with trained personnel. The RT-PCR procedure also takes a long time and has complex steps [11]. Therefore, testing samples in mass quantities is difficult to realize. In this case, methods that are simple, fast, accurate, and sensitive are really needed to deal with the spread of the virus in the future. SPR biosensors have been used for more than 30 years to rapidly and accurately measure several biological and chemical species with very low detection limits at the atto- or femtomolar order [12]. By utilizing surface plasmon waves (SPWs), the interaction between the receptor molecule and the analyte detected can be monitored in real-time by observing the sensorgram signal. This device also offers parallel analysis through the development of surface plasmon resonance imaging (SPRi) to offer more complete information [13].

**Figure 1:**
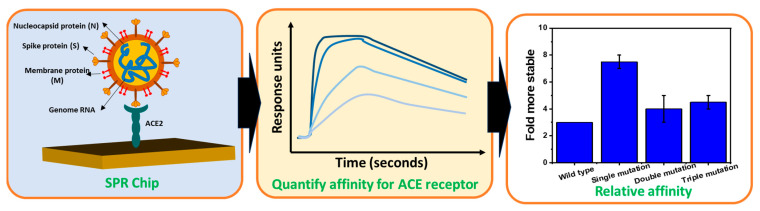
Schematic diagram to illustrate the contents of this paper.

Several research groups have reviewed topics related to the use of SPR technology for dealing with COVID-19. For detection, diagnosis, and early screening of SARS-CoV-2, this topic has been reviewed by Pandey’s group [14] and Nor’s group [15]. More specific topics, such as using SPR technology to find materials that inhibit the entry of SARS-CoV-2 have been reviewed by Mauriz’s group [16]. This paper tries to review the different perspectives on the use of SPR in dealing with the COVID-19 pandemic. The main points that will be presented in this paper are illustrated in Figure 1. This paper begins with an introduction to the principles of SPR technology and continues with an introduction to the kinetic parameters of SPR signals. Studies show that the SARS-CoV-2 spike protein will bind to ACE2 to mediate the entry of the virus into cells [17]. Therefore, in the last part of this paper, we will only focus on reviewing kinetic parameters in several cases of SARS-CoV-2 mutation with ACE2. ACE2 is the main natural receptor that mediates viral entry, and therefore, no modification of ACE2 is required in studying the kinetic parameters of different mutation and variants of SARS-CoV-2 [18]. Furthermore, information related to kinetic parameters will be useful in monitoring epidemiological developments, vaccine development, drug discovery, and so on [16,19].

## 2. Working Principle and Development of a Prism-Based SPR Biosensor

The SPR biosensor is one type of biosensor that utilizes surface plasmon waves in its work. Surface plasmon waves are generated when light interacts with free electrons at the interface between a metal (or other conducting material) and a dielectric (usually glass, air, or liquid) [20]. When monochromatic light with p-polarization strikes a metal surface, the light will be absorbed by electrons and cause collective oscillations of electrons called plasmons [21]. Surface plasmon waves propagate on metal and dielectric surfaces with a wave number symbolized by $k_{sp}$ and its magnitude can be determined mathematically using the following equation [22]:

(1) $$k_{sp}=\frac{\omega }{c}\sqrt{\frac{\left(\varepsilon_{m}\varepsilon_{d}\right)}{\left(\varepsilon_{m}{+\varepsilon }_{d}\right)},}$$

where ω is the frequency of incident light, c is the speed of light, $\varepsilon_{m}$ and $\varepsilon_{d}$ are the dielectric constants of the metal and the dielectric, respectively. Because the dielectric constant is related to the refractive index (*n*) based on the relationship $\sqrt{\varepsilon_{real}}$ [23], then $k_{SP}$ in Equation (1) can be modified to [24]:

(2) $$k_{sp}=\frac{\omega }{c}\sqrt{\frac{\left({n_{m}}^{2}\;{n_{d}}^{2}\right)}{\left({n_{m}}^{2}\;{{+n}_{d}}^{2}\right)},}$$

where $n_{m}$ and $n_{d}$ indicate the refractive index of the metal and the dielectric.

**Figure 2:**
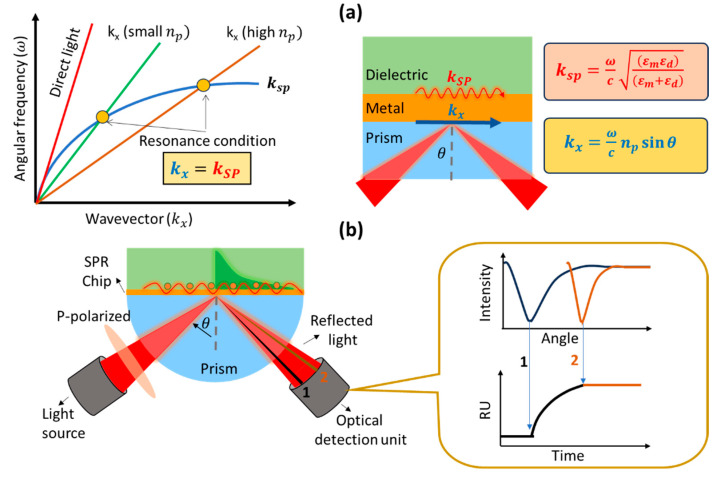
(**a**) Surface plasmon dispersion curve. (**b**) Schematic of the prism-coupled SPR biosensor and the resulting signal.

If we measure the intensity of reflected light, at a certain angle of incidence we will find an angle where the light will show a very low intensity. This happens because the incident light is completely absorbed by the electrons. The angle at which the intensity of the reflected light shows the smallest intensity is usually called the SPR angle or the resonance angle. The resonance condition occurs when the wave number of the photon is equal to the wave number of the surface plasmon. This can be explained using a dispersion curve as shown in Figure 2a. Surface plasmon waves cannot be excited by direct light because the wave vector of the surface plasmon is higher than the incident light. The wave vector of the surface plasmon (blue curve) will never intersect with the wave vector of the photon (red curve) over the entire wave number range. To achieve resonance conditions, the dispersion curve of the surface plasmon must be reduced or the dispersion curve of the photons increased. One widely used approach is to add a prism with a high refractive index to increase the photon dispersion curve. By adding a prism, the photon wavenumber changes from

(3) kx=ωc sin⁡θ

to

(4) kx=ωc np sin⁡θ.

Since the refractive index of the prism is constant, the resonance angle will depend on the presence or absence of absorbed molecules at the metal and dielectric interfaces on the sensing surface. The presence of absorbed molecules will result in a shift in the resonance angle. Therefore, we can monitor the presence or absence of adsorbed molecules or find out how fast the molecules are absorbed (kinetic analysis) from the SPR sensorgram (Figure 2b). There are several parameters that we can obtain from the SPR sensorgram, which are the association rate constant (k_a_), dissociation rate constant (k_d_), equilibrium association constant (K_A_), and equilibrium dissociation constant (K_D_). All these quantities will be discussed in the next section.

In 1982, SPR technology was used for gas detection. SPR technology continues to be developed for wider applications such as food safety [25], environmental monitoring [26], medical diagnosis and detection [27], drug discovery [14], and others. Apart from these, another focus in the development of SPR biosensors is on transducer engineering to achieve sensitive sensors so that analytes can be detected down to the smallest possible concentration. By modifying the SPR chip using 2D materials such as graphene and MoS_2_, various biomarkers with very small concentrations can be detected. Chiu et al. modified the surface of a thin layer of gold on an SPR chip with graphene oxide to detect human chorionic gonadotropin (hCG) proteins [28]. The detection limit that can be achieved by this sensor system is 0.065 nM, and when compared with conventional SPR biosensors, modification with graphene oxide can increase the sensitivity of the biosensor up to 16 times higher. In 2021, Chiu et al. also modified the SPR chip using MoS_2_ to detect pregnancy-associated plasma protein-A2 (PAPP-A2) [29]. The detection limit obtained was 0.05 pg/mL with a linear range of detection ranging from 0.1 to 1100 pg/mL.

## 3. Principle of SPR Kinetics

An SPR biosensor is a potential tool which can be used to investigate phenomena that occur on the sensing surface. Surface phenomena such as molecular absorption cause changes in the refractive index and change the SPR angle. The SPR angle will shift to a higher angle when a molecule is adsorbed and will shift to a smaller angle when a molecule is released from the sensing surface. The response of the SPR biosensor due to a phenomenon on the sensing surface can be plotted at any time in real-time, and the resulting curve is called a sensorgram curve. Kinetic parameters that describe bonding events can be obtained, such as the association, dissociation, and equilibrium constants [30].

**Figure 3:**
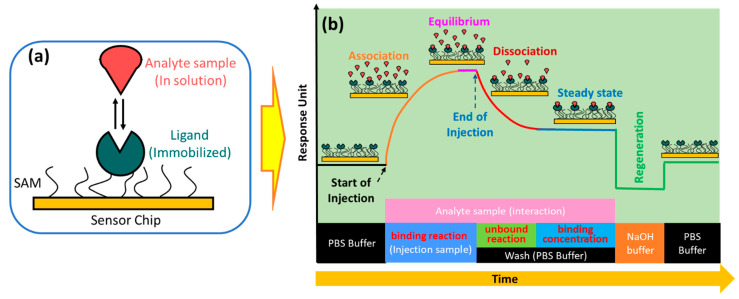
(**a**) Biosensor chip architecture; (**b**) SPR sensorgram at different phase.

In the simplest SPR experiment, the experiment begins with the immobilization of the active ligand to specifically recognize the molecule to be detected (Figure 3a). Ligands can be immobilized on the sensor surface through a material that is usually called self-assembled monolayers (SAM) [31]. The target molecule to be detected in this case is called the analyte. The buffer that flows over the sensor surface is called the running buffer [32]. It is essential to condition the sensor surface with an appropriate buffer solution, and the types of buffers that are widely used in SPR experiments are HEPES, Tris, or PBS [33].

As shown in Figure 3b, the capture of the analyte by the ligand begins with the conditioning of the running buffer signal. The signal on the sensorgram must form a stable baseline. External influences that cause signal fluctuations in the sensorgram, such as temperature, must be minimized to be as small as possible [34]. Once a baseline is obtained, the solution containing the analyte can be injected. Ligands immobilized on the sensor surface will capture the analyte, and this is indicated by an increase in the sensorgram signal. The magnitude of the sensorgram signal increase depends on the number of active ligands. After the active ligand pairs with the analyte, the sensorgram signal will be in an equilibrium state. This phase is called the association phase. In the same phase, non-specific interactions caused by the presence of impurities in the analyte solution are also very likely to occur. Therefore, washing is carried out using a running buffer. The remaining analytes and components that are not tightly bound will be removed from the sensor surface. This phase is called the dissociation phase. After this phase has been successfully completed, the sensorgram signal will show a steady state. Finally, the regeneration solution is injected into the sensing surface to break the bond between the analyte and the ligand. If the ligands are immobilized properly, all ligands will remain on the sensing surface, and measurements for other analyte samples can be made using the same SPR chip [30,35,36,37]. SPR biosensors are usually equipped with two channels: where the first channel is used to obtain a sensorgram signal from the analyte, and the other channel is used to obtain a reference sensorgram signal. The actual signal is obtained after correction by subtracting the measured analyte signal from the reference signal [38,39].

If the ligand on the sensing surface is symbolized by *B* is bonded to the analyte symbolized by *A*, the bond between them produces a complex molecule symbolized by *AB*. The interaction can be written by the following equation [37]:

(5) $$A+B\overset{ka,kd}{↔}AB$$

The association rate constant is defined as the number of complex molecules formed per unit time at concentrations of *A* and *B*. This quantity is usually denoted by $k_{a}$ and in some references it is denoted by $k_{on}$. Furthermore, the dissociation rate constant indicates the number of complex molecules that decay over time. This quantity is usually symbolized by $k_{d}$ and in some references it is written as $k_{off}$. Equilibrium is reached when the rates of association and dissociation are equal. The association and dissociation equilibrium constants represent the affinity of the interaction between ligand and analyte. The affinity of the molecule for association is expressed by *K_A_*. The last one is *K_D_*. This quantity indicates the stability of the formation of the *AB* complex molecule where a high *K_D_* value indicates the low stability of the formation or interaction of the *A* and *B* molecules [40]. Table 1 below shows the definitions, units, and typical ranges for $k_{a}$, $k_{d}$, $K_{A}$, and $K_{D}$.

To produce a good sensorgram signal, there are a few tips to consider. Some of them are related to pH and the type of buffer used, the reactive group, and the molecular weight. The reactive group is important because it ensures covalent coupling of the ligands on the sensor surface. Ligands must contain reactive groups such as $-{NH}_{2},-SH,$ or −COOH to capture proteins and oligonucleotides. Molecular weight will affect the signal that will be generated, where smaller molecules can change the refractive index to be lower than that of larger molecules. In many cases, researchers usually immobilize a molecule with a smaller molecular weight as a ligand to obtain a higher signal [41]. The relationship between the molecular weight of the analyte ($MW_{analyte}$), ligand ($MW_{ligand}$), and the ligand response ($R_{ligand}$) with the binding capacity of the analyte is shown in the following equation [42]:

(6) $$Analyte\;binding\;capacity\;(RU)=\frac{MW_{analyte}}{MW_{ligand}}\times R_{ligand}(RU)$$

The response to the binding capacity of the molecule is only maximal when the ligand on the sensing surface is fully active. However, in many experiments, some ligands on the sensing surface are not active, so the signal response obtained is smaller.

## 4. COVID-19 Virus and Its Mutation

**Figure 4:**
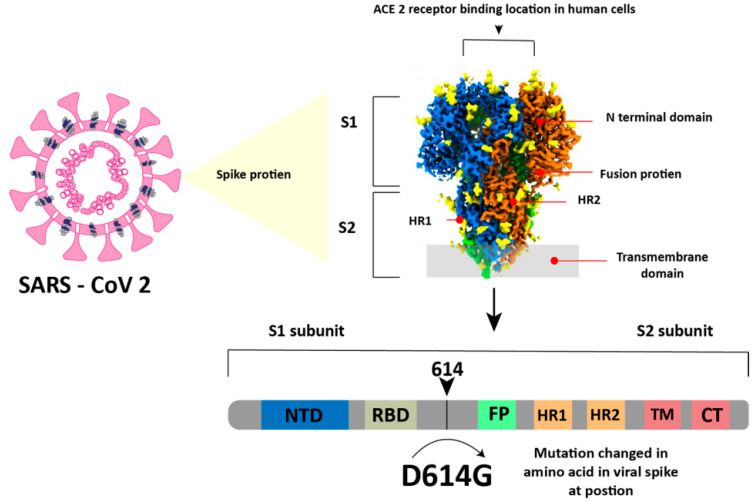
Structure of the SARS-CoV-2 virus. Reproduced with permission from [45]. Copyright (2023), Springer.

The SARS-CoV-2 virus is a member of the betacoronavirus genus and has a genome similar to that of SARS-CoV (about 80%) and Middle East respiratory syndrome coronavirus (MERS) (about 50%) [43]. In simple terms, this virus has a spherical shape with a diameter of 130 nm and is surrounded by a spike-like structure on its entire surface, as shown in Figure 4. This virus encodes sixteen non-structural proteins (NSPs) and four structural proteins, which are the nucleocapsid protein (NP), spike glycoprotein (SP), membrane protein (MP), and envelope protein (EP) [44]. The SP is composed of an N-terminal S1 subunit and a C-terminal S2 subunit located near the membrane [45]. The S1 subunit contains RBD, which can bind to ACE2 as a cellular receptor during virus entry, and after that, the Transmembrane domain in subunit S2 will help the virus enter the host cell [44,45].

SARS-CoV-2 continues to evolve over time. These changes can affect the characteristics of the virus, such as changes in the speed of its spread. Since it was first identified in December 2019 to November 2021, SARS-CoV2 has mutated five times with variants called alpha B.1.1.7 in September 2020, beta 1.351 in October 2020, gamma P.1 in November 2020, delta B.1.617.2 in December 2020, and the last one is Omicron B.1.1.529 in November 2021. Before the discussion continues, it is very important to know the difference between a mutation and a variant to make the discussion clearer and to avoid misunderstanding. Mutations are defined as amino acid exchanges (nonsynonymous or missense) in spike glycoproteins, while other nucleotide changes (synonymous or non-missense) are defined as variants [46,47]. One of the most significant mutations in SARS-CoV-2 is the D614G mutation (Figure 4). In the D614G mutation type, the amino acid aspartate (D) at position 614 in the viral spike protein is replaced by the amino acid glycine (G) [48,49].

**Figure 5:**
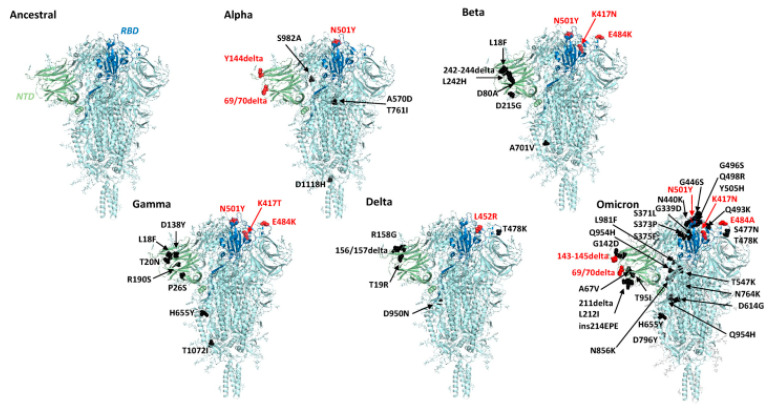
3D structure of the spike protein of SARS-CoV-2 variants alpha, beta, gamma, delta, and omicron in comparison with an ancestral virus. Reproduced with permission from [50]. Copyright (2023), mBio.

Mutations in the SARS-CoV-2 virus can occur in various positions in the viral genome. The SARS-CoV-2 genome is a long RNA chain, and mutations can occur in the spike protein, RBD, N-Terminal Domain (NTD) and others. The complete amino acid changes that occur as a result of the mutations are shown in Figure 5, and to illustrate the differences between each variant, we compared them to the structure of the ancestral protein. In addition, RBD is a part of the S protein that interacts directly with the ACE2 receptor in human cells. Mutations in the RBD can affect the virus’ ability to bind to human cells. Therefore, we show the mutations that occur in the RBD which are shown in Table 2.

## 5. Application of SPR Technology for SARS-CoV-2 Detection and Analysis of Its Binding

Biosensors are detection devices that use ligands as molecular recognition elements to detect the target molecule specifically. In the context of detecting the COVID-19 virus, several materials can be used as ligands, such as ACE2 [52], monoclonal antibodies (Mabs) [53], aptamers [54], peptides [55], and immunoglobin molecules (IgM/IgG) [56]. Table 3 below summarizes the various nanophotonic biosensors that have been developed, and to illustrate sensor performance, we include the limit of detection (LoD) obtained from each sensor.

Of the ligands mentioned above, ACE2 is a type of ligand that is widely used to detect the SARS-CoV-2 virus. ACE2 is a natural receptor that is available in various tissues in the human body, especially in cells in the respiratory tract (highest in the olfactory bulbs) [65]. SARS-CoV-2 uses the spike protein on its surface to bind to the ACE2 receptor on human cells. By utilizing this type of ligand, the binding affinity of different variants of SARS-CoV-2 resulting from mutations can be analyzed and identified based on changes in their kinetic parameters. This information is very important not only in virus detection, but also in vaccine development and drug discovery.

Although ACE2 has been identified as the main receptor that mediates entry of the SARS-CoV-2 virus into human cells, heparan sulfate has been identified as a molecule that plays a role in the early stages of viral infection in host cells. A comparison of the affinity between heparin and ACE2 with spike protein has been investigated previously by Liu et al. [66]. They started the experiment by immobilizing heparin and ACE2 on the surface of a streptavidin chip. After that, the $K_{D}$ value was determined on three different protein samples, namely RBD, spike monomer, and spike trimmer. The analysis results show that the $K_{D}$ value of the interaction between ACE is always smaller than heparin. In the case of the RBD protein, the $K_{D}$ values for the heparin chip and ACE2 were ~1000 nM and 3.6 nM, respectively. These results confirm that the RBD domain has a much higher affinity for ACE2 compared with heparin.

**Figure 6:**
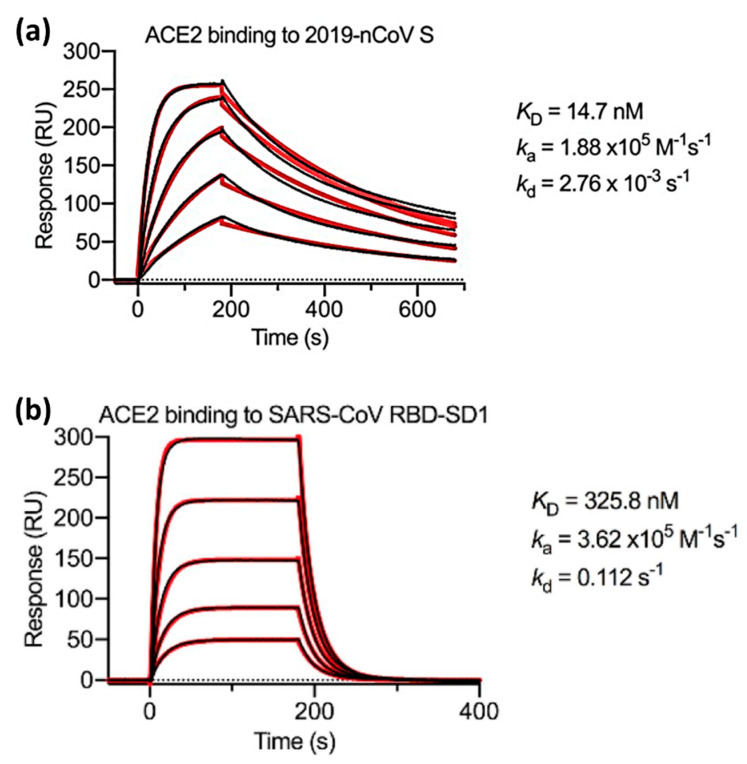
SPR sensorgram showing kinetic binding for ACE2 with (**a**) 2019-nCoV S, (**b**) SARS-CoV RBD-SD1. Reproduced with permission from [67]. Copyright (2020), Science.

Previous results showed that the affinity of ACE2 was much higher than that of heparin. Therefore, Wrapp et al. used ACE2 and compared $k_{a}$, $k_{d}$, and $K_{D}$ on two different samples which were s-proteins of the novel coronavirus (2019-nCoV) and the RBD sub-domain 1 (SD1) of SARS-CoV. Serial dissolution of ACE2 was carried out to obtain a 1:1 binding stoichiometry. After ACE2 injection, a sensorgram for each concentration of ACE2 was obtained, as shown in Figure 6. The black line shows the real data while the red line shows the fitting data. If we compare the $K_{D}$ value of 2019-nCoV S and SARS-CoV RBD-SD1, the $K_{D}$ value of SARS-CoV RBD-SD1 shows a much higher value of 325.8 nM. As explained in the previous section, a high $K_{D}$ value indicates a low stability in the formation of bonds between the two molecules. Therefore, it can be concluded that 2019-nCoV S has a higher affinity, which is 20 times higher than the binding between ACE2 and SARS-CoV RBD-SD1 [67].

**Figure 7:**
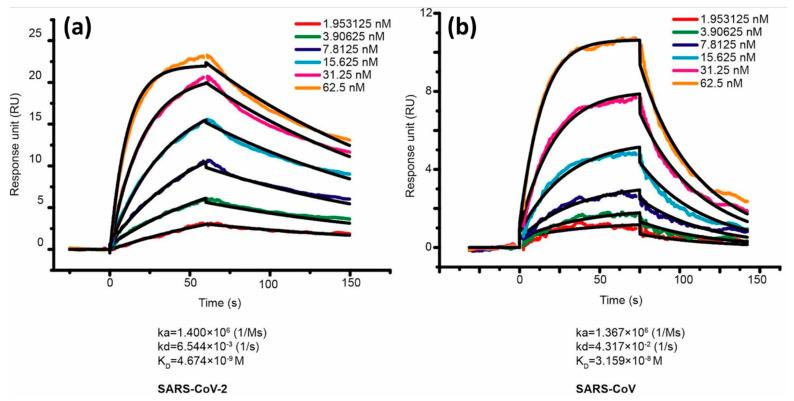
Binding curves of immobilized human ACE2 with the SARS-CoV-2 RBD (**a**) and SARS-CoV RBD (**b**). Reproduced with permission from [43]. Copyright (2020), Nature.

In the same year, Lan et al. also compared the binding affinity between SARS-CoV-2 RBD and SARS-CoV RBD. ACE2 was employed as a ligand and immobilized on the CM5 chip sensor surface. The response generated after ACE2 injection is 500 response units. Serial dilution was carried out on samples of SARS-CoV-2 RBD and SARS-CoV RBD to obtain a 1:1 bonding model using Biocore Insight Software evaluation software (GE Healthcare, Massachusetts, United States) and the concentrations obtained were in the range of 1.95 nM to 62.5 nM. Figure 7 shows a sensorgram for this concentration range where the $K_{D}$ values of SARS-CoV-2 RBD and SARS-CoV RBD were 4.7 nM and 31 nM, respectively [43]. Walls et al. in 2020 conducted a kinetic analysis between human ACE2 (hACE2) with SARS-CoV-2 S and SARS-CoV S using a biosensor based on biolayer interferometry (BLI). The results obtained showed that the $K_{D}$ values of SARS-CoV-2 S and SARS-CoV S were 1.2 nM and 5 nM, respectively [68]. If we compare some of the results above, the $K_{D}$ values obtained show a slightly different magnitude, but both have similarities, namely the $K_{D}$ of SARS-CoV-2 S is always smaller than that of SARS-CoV S. The conclusion from our discussion above shows that the kinetic data prove that SARS-CoV-2 S has a higher binding affinity to the ACE2 receptor.

To date, the SPR biosensor has been proven to be able to be used to identify mutation phenomena in the SARS-CoV-2 protein. The effect of D614G on the kinetic parameters of SARS-CoV-2 S proteins and ACE2 has been successfully investigated by Yurkovetskiy et al. [69]. In this research the SPR biosensor was designed to distinguish between G614 and D614. A comparison of kinetic parameters between the SARS-CoV-2 D614G S protein and the ancestral protein was also carried out. The results of this study indicate that the detected RBD mutation has a correlation with SARS-CoV-2 infectivity. The SARS-CoV-2 variant that continues to mutate causes the infectivity of the virus to enter the body to be higher. Several studies have reported changes in the binding affinity of ACE2 after the virus mutates [49]. Xue et al. investigated nine different mutations and compared them with wild type (WT). The mutants investigated were Q498W, Q498R, T500W, S477H, Y505W, T500R, N501V, Y489W, and Q493M [70]. The $K_{D}$ value of WT is 21.08 nM. Of the nine mutants investigated, three of them had higher $K_{D}$ values, namely T500W ($K_{D}$ = 21.8 nM), N501V ($K_{D}$ = 158.50 nM), and Y489W ($K_{D}$ = 38.90 nM). Since most of the $K_{D}$s decreased after mutation (the smallest $K_{D}$ was Q493M (6.9 nM)), this indicates that the presence of viral mutations could strengthen the binding affinity.

**Figure 8:**
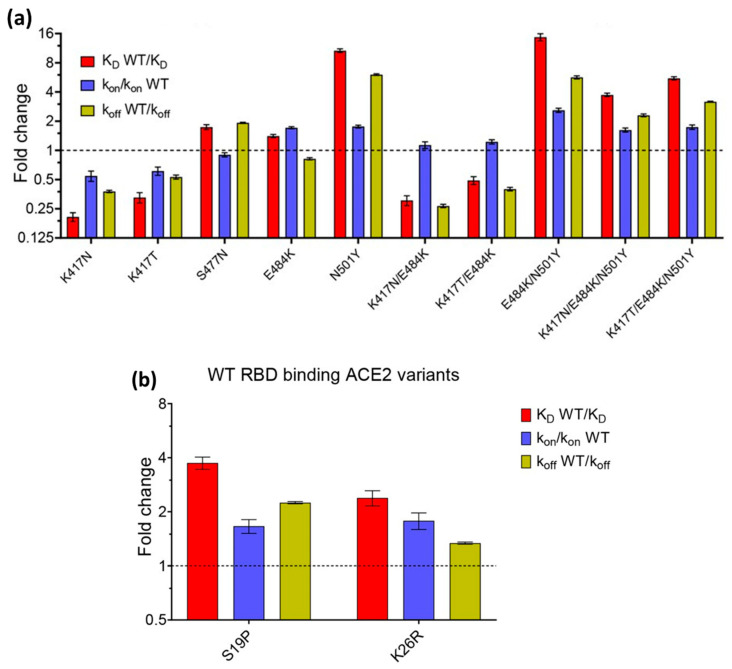
Kinetic parameters (*k_a_*, *k_d_*, and *K_D_*) due to (**a**) RBD mutation and (**b**) ACE2 mutation. Reproduced with permission from [71]. Copyright (2020), eLife.

The effect of mutations was also investigated by Barton et al. in 2021 [71]. They investigated the affinity and kinetics of five types of RBD mutations (K417N, K417T, N501Y, E484K, and S477N) and two ACE2 mutations (S19P and K26R). Then, they compared it with WT RBD (In Figure 8, the affinity and kinetics of WT RBD are shown by dashed lines). As shown in Figure 8a, the RBD mutation increased binding to the single mutations (S477N, E484K, and N501Y). Of these three single-mutation types, N501Y showed the highest increase, which was 10 times higher than WT RBD. Not only single mutations, but also double (E484K/N501Y) and triple mutations (K417N/E484K/N501Y and K417T/E484K/N501Y) have higher affinity than RBD WT. The same results also occur in ACE2 mutations. Of the two types of ACE2 mutations investigated, both increased the binding affinity between ACE2 and RBD.

Not only can changes in the binding affinity parameters of the SARS-CoV-2 virus caused by mutations be predicted through experimental studies, but they can also be predicted through computational studies. Various approaches to this problem have been developed, including Free Energy Perturbation (FEP) [72,73], machine learning [74], statistical potentials [75], and various force field-related scoring functions embedded in programs such as FoldX [76] and Rosetta [77]. Sergeeva et al. investigated the effect of mutations on the binding affinity of ACE2 with SARS-CoV-2 using FEP [78]. In this study, they investigated the binding affinity (KD) of ACE2 with wild type (WT) and 23 single mutants. In addition, the epistatic effect of the Q498R N501Y double mutant on the omicron variant can also be determined accurately. These computational results have been successfully confirmed based on SPR experiments.

Makowski and his colleagues have analyzed the mutational variations in SARS-CoV-2 against ACE2 using a computational approach supported by machine learning [79]. In this study, it was found that the affinity ($K_{A}$) of the beta (RBD Mutations: K417N, E484K, N501Y) and gamma (RBD Mutations: K417T, E484K, N501Y) variants tended to maintain their affinity level compared to the wild type (WT), remaining at around 1 × 10^11^ M. However, the affinity ($K_{A}$) of the alpha (RBD Mutation: N501Y), epsilon (RBD Mutation: L452R), and delta (RBD Mutation: L452R, T478K) variants experienced a significant increase, reaching 1.48 × 10^11^ M for the alpha variant, 2.54 × 10^11^ M for the epsilon, and 4.37 × 10^11^ M for the delta variant. This study also investigated the individual impact of 15 RBD mutations on ACE2 affinity. It was found that five RBD mutations (G339D, N440K, S477N, T478K, and N501Y) increased the affinity of ACE2. In addition, most RBD mutations are also predicted to increase antibody release, with the E484A mutation shown to substantially decrease the neutralization activity of human convalescent serum.

Affinity is information that refers to the strength of an interaction between a particular molecular target and a receptor in a human cell. From the references discussed above, changes in the structure of the virus and the mutations that occur result in changes in its binding affinity. Understanding these affinity changes is critical for controlling viral transmission, vaccination strategies, and future drug development. In the context of biosensors, machine learning is not only used to obtain affinity data from SPR biosensors. Machine learning has also been utilized in studying different nonlinear optical effects. The combination of machine learning analysis tools and multipotonic effects has enormous potential in data interpretation. Gupta et al. used Principal Component Analysis (PCA) as a learning machine to improve SERS signals [80]. The use of PCA in this research was able to increase the SERS signal to be up to three times higher. Other researchers, namely Paryanti et al. [81] and Williamson et al. [82], use a different algorithm which is Neural Networks. Details regarding the use of machine learning in nonlinear optical effects are discussed in the following paper [83].

Martinez et al. summarize the process in machine learning into several parts [83]. After the biosensor data are acquired, the raw data obtained are first processed through data filtering and segmentation. Furthermore, normalization is also carried out at this stage to homogenize the scale or data type according to the data’s properties. After that, the appropriate model must be determined and this depends on the problem being analyzed and the characteristics of the data being analyzed. There are three models that can be used, namely classification, regression, and clustering. After the model is selected, model learning and evaluation is carried out. Once the selected model is valid and successfully implemented, regular monitoring and maintenance is required to update the algorithm according to changes within the data or environment. In this way, hidden patterns of complex biological systems such as SARS-CoV-2 mutations can be recognized with the help of computing systems in machine learning. The use of machine learning in analyzing biosensor data will be very important in dealing with future pandemics.

## 6. Conclusions and Future Prospective

The global COVID-19 epidemic has become a major threat to public health worldwide. Thousands of mutations have been identified in the SARS-CoV-2 genome. This paper was written to summarize the dynamics of SARS-CoV-2 mutations using SPR biosensors. The main advantage of SPR biosensors is their ability to offer real-time monitoring of molecular interactions. This property is very important, especially in the case of studying the structure and dynamics of viruses. From the information previously described, we know how the interaction affinity between ACE2 and SARS-CoV-2 changes, both wild type and mutated. Deep insights into interaction dynamics can be explored in more depth by analyzing levels of association and dissociation.

SPR biosensor is a type of label-free biosensor. The absence of labels (e.g., fluorophores) minimizes the complexity in the sample preparation and the risk of contamination. The analysis process becomes faster, and the measured signal is a real signal that represents the properties of the analyte itself. The progress that has been achieved to date is that SPR biosensors can be used to detect analytes up to the atto order. Another advantage of the SPR biosensor is that the transducer is also compatible for use simultaneously with other biosensors. As a result, it is possible to obtain dual mode sensor. Regarding the dual mode biosensor, the SPR biosensor has been successfully integrated with electrochemical [84], surface-enhanced Raman scattering (SERS) [85], electrolyte-gated field-effect transistor (EG-FET) [86].

From the advantages mentioned above, currently existing biosensors, especially SPR biosensors coupled to prisms, are still bulky in size. Therefore, to obtain a more compact and portable device, several research groups are developing fiber-optic-based SPR biosensors. To obtain better sensitivity and lower detection limit, several structures have been developed such as single-mode optical fibers (unclad, side-polished, tapered, and U-shaped), long period fiber gratings (LPFG), tilted fiber Bragg gratings (TFBG), and specialty fibers (plastic or polymer, microstructured, and photonic crystal fibers) [87]. Furthermore, to simultaneously detect several targets or biomarkers in one test, multiplex biosensors need to be developed. This is very important to obtain more comprehensive data. By combining multiplexing capabilities with high detection sensitivity, multiplex biosensors enable more effective monitoring of new variants and mutations that may emerge over time.

## Figures and Tables

**Table 1:** Definition, units, and typical range of $k_{a}$, and $K_{D}$.

	$k_{a}$	$k_{d}$	$K_{A}$	$K_{D}$
Definition	A+B→AB	AB→A+B	$\frac{\left[AB\right]}{\left[A\right]\left[B\right]}=\frac{k_{a}}{k_{d}}$	$\frac{\left[A\right]\left[B\right]}{\left[AB\right]}=\frac{k_{d}}{k_{a}}$
Unit	$\frac{L}{mol\;s}$	$s^{-1}$	L/mol	mol/L
Typical range	10^3^ − 10^7^	10^−1^ − 5 × 10^−6^	10^5^ − 10^12^	10^−5^ − 10^−12^

**Table 2:** Variants of concerns’ lineages, and the location of their mutations on the RBD [51].

Variants	Lineage	Mutations on the RBD
Alpha	B.1.1.7	N501Y
Beta	B.1.351	K417N, E484K, N501Y
Gamma	P.1	K417T/N, E484K, N501Y
Delta	B.1.617.2	L452R, T478K
Omicron	B.1.1.529	G339D, S371L, S373P, S375F, K417N, N440K, G446S, S477N, T478K, E484A, Q493R, G496S, Q498R, N501Y, Y505H

**Table 3:** Representative paper for nanophotonic biosensors for detecting SARS-CoV-2.

Optical Effect	Ligands	Analytes	LoD	Ref.
SERS	ACE2	SARS-CoV-2 S Protein	0.1 fg/mL	[57]
LSPR	Aptamer	SRBD and SARS-CoV-2 pseudovirus	21.9 pM	[58]
SPR	anti-SARS-CoV-2 spike S1 protein	S1 protein	12 fg/mL	[59]
SPR	anti-SARS-CoV-2 spike MAbs	SARS-CoV-2 spike antigen	0.08 pg/mL	[60]
Fiber optics	ACE2	SARS-CoV-2 spike protein	∼3.05 ng/mL	[61]
SERS	SARS-CoV-2 spike antibody	Spike protein	0.77 fg/mL	[62]
SERRS	SARS-CoV-2 antibodies	SARS-CoV-2 spike glycoprotein (S1)	0.60 pM	[63]
BLI	ACE2	S protein	500 pg/mL	[52]
Fiber optics	single-stranded DNA (ssDNA) aptamer	SARS-CoV-2 Nucleocapsid Protein	2.8 nM	[64]

Note: SERRS: Surface-enhanced Resonance Raman scattering; BLI: Biolayer interferometry.

## Data Availability

Not applicable.
